# Frameworks to Evaluate the Quality of Public Involvement in Health and Social Care Research: A Scoping Review

**DOI:** 10.1111/hex.70666

**Published:** 2026-05-16

**Authors:** Rose Hutton, Alice Moult, Dereth Baker, Anthony A. Fryer, Andrew Meakin, Opeyemi Babatunde

**Affiliations:** ^1^ Impact Accelerator Unit, School of Medicine Keele University, Keele Staffordshire UK; ^2^ Expert Citizens Community Interest Company, Stoke‐on‐Trent Staffordshire UK

## Abstract

**Introduction:**

Public involvement (PI) in health and social care research is well established. However, questions remain about the quality of PI activities and the consistency with which high‐quality PI is conceptualised. This lack of clarity in how PI quality should be defined and assessed creates challenges for identifying and promoting best practice. While many tools and guidelines exist to support researchers to involve public contributors, it is unclear how many specifically address the evaluation of quality. This scoping review aimed to identify and compare existing frameworks developed to assess the quality of PI in health and social care research.

**Methods:**

A scoping review was conducted in line with the Arksey and O'Malley framework. Nine electronic databases were searched for articles published from 2000 onwards. In addition, Google and the websites of large health and social care charities and research councils were searched to identify unpublished (grey) literature. Forward and backward citation tracking was used to identify sources describing the development of evaluation frameworks to ensure sufficient information on the frameworks was available.

**Results:**

From the search, we identified six frameworks designed to evaluate the quality of PI in health and social care research. These were: CUBE, INSIGHT, PiiAF (Public Involvement Impact Assessment Framework), PEQG (Patient Engagement Quality Guidance tool), QIF (Quality Involvement Framework), and PIRIT (Public Involvement in Research Impact Toolkit). While all the frameworks share a common goal to promote best practice in PI, they differ in their conceptualisations of quality, approaches to evaluation, and intended role in the research cycle.

**Conclusion:**

This review provides the first comparative analysis of evaluation frameworks focused on PI quality and offers practical guidance for framework selection. Several evaluation frameworks are available for researchers and public contributors to assess the quality of involvement activities. Because the frameworks identified varied in their conceptualisation of quality and approaches to evaluation, research teams must consider which framework is most aligned with their evaluation goals.

**Patient or Public Contribution:**

A public contributor was involved in the preparation and review of the manuscript, approved the final version, and is included as a co‐author. Their contribution included a retrospective review of the study design, inclusion and exclusion criteria, and interpretation of the findings. Due to the absence of funding at the outset of the project, public contributors were not involved in the initial study design or scoping review.

## 1 Background

Public involvement (PI) can be defined as research carried out ‘with’ or ‘by’ patients and public contributors rather than ‘to’, ‘about’, or ‘for’ them [1]. In practice, this involves partnering with members of the public to seek their input and expertise on various aspects of research. This can include research priority setting, inputting on research design and methodology, interpreting data, and advising on dissemination activities. The value of high‐quality PI can include increased research relevance and improved research quality, as well as benefits for the public contributors such as enhanced public voice.

Despite the widespread nature of PI in health and social care research, questions have been raised about the quality of some PI activities. Similarly, the consistency of PI quality has also been called into question, with PI activities in primary care research, for example, varying in quality across research designs and topics [2]. Variation in the context and approaches to PI, combined with the lack of frameworks that explicitly evaluate quality, creates challenges in identifying best practice.

The international literature on how to evaluate PI has more than tripled in recent years [3]. Researchers have conducted systematic reviews, realist evaluations, and qualitative studies to explore stakeholder views. Practical guidance for incorporating the evaluation of PI exists, and at least 65 frameworks have been developed to assess the nature of PI in research [4]. Despite recognition of the importance to evaluate PI, there remains a lack of clarity around how PI *quality* should be conceptualised and assessed. While guiding principles such as the UK Standards for Public Involvement (UKSPI) encourage researchers to reflect on the quality of PI [5], without explicitly defining and conceptualising what quality is, evaluation may not be consistent across activities. This inconsistency could undermine the credibility of evaluation efforts.

To evaluate the quality of PI, greater insight is needed in relation to which frameworks conceptualise quality, and the variation in how they approach evaluation. Doing this would improve understanding of PI best practice as well as highlighting the similarities and differences between frameworks. This would, in turn, help researchers to conduct effective evaluations of the quality of their own PI activities, thereby supporting continuous improvement.

Whilst there have been previous reviews on PI evaluation frameworks [3, 4, 6], these have either focused broadly on evaluation tools or examined frameworks without specifically analysing how quality is conceptualised and assessed. To date, no review has specifically identified and compared frameworks designed to evaluate the quality of PI in health and social care research. To address this gap, this scoping review aimed to: (a) identify the frameworks available for evaluating PI quality, and (b) compare and contrast their approaches to evaluation and their conceptualisations of quality. Our aim is that this will support research teams and research‐active organisations in selecting and applying frameworks most appropriate to their context.

## 2 Methods

### 2.1 Overview

As the purpose of this review was to identify and compare frameworks aimed at evaluating PI quality, rather than to synthesise evidence, a scoping review approach was chosen over a systematic review. Formal critical appraisal of the included studies was therefore not undertaken, as the focus of the review was to map and explore frameworks within the existing literature rather than to evaluate methodological quality or effectiveness. The five‐step framework for scoping reviews by Arksey and O'Malley [7], refined by Levac et al. [8] was followed. The steps are:

1. Identify research questions.
2. Searching for relevant studies.
3. Selecting studies.
4. Charting the data.
5. Collating, summarising, and reporting the results.

These steps were used to develop a protocol for the review and to report how the review process was conducted. Reporting was also guided by the Preferred Reporting Items for Systematic Reviews and Meta‐Analyses (PRISMA) extension for scoping reviews [9]. The following research questions were developed by the research team to guide the review:

- What frameworks exist for evaluating PI quality in health and social care research?
- What are the strengths and weaknesses across approaches?
- How do they conceptualise quality?

### 2.2 Identifying Relevant Studies

A systematic search strategy was developed by the research team. Searches were conducted in nine databases (CINAHL, PsycINFO, MEDLINE, Web of Science, PubMed, ASSIA, Academic Search Complete, AMED, and Sociology Database) in April 2024. The search was limited to work published from January 2000 onwards. The year 2000 was chosen as the cut‐off point to ensure both feasibility and relevance of the included literature.

**Figure 1:**
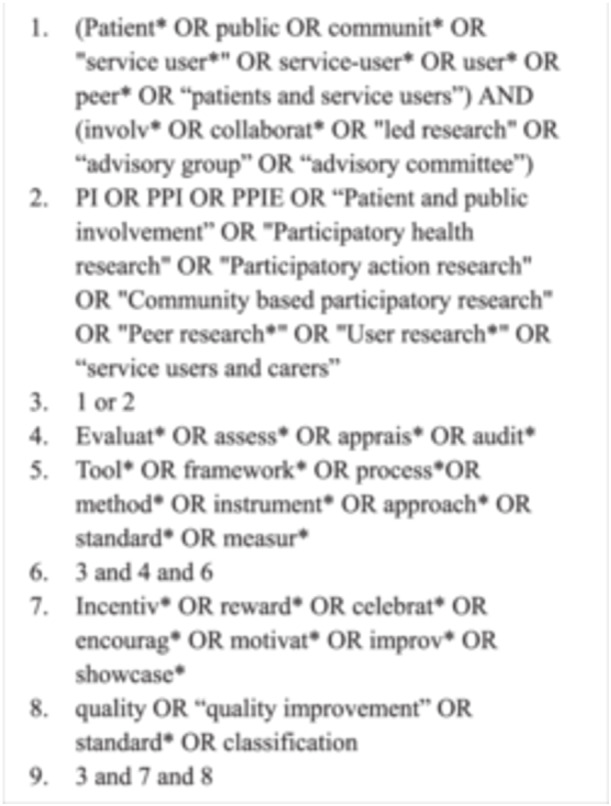
Search terms.

Due to the variety of terminology used by researchers to describe PI, an inclusive approach was taken when developing the search terms (Figure 1). After discussion, ‘public engagement’ was not included as a keyword. Public engagement refers to the sharing of research with the public, rather than actively collaborating with them as partners [1]. Although the terms are sometimes used interchangeably, this review specifically aimed to identify and examine frameworks for evaluating the quality of PI, rather than engagement.

To identify grey literature, we searched for reports, policy documents, non‐peer‐reviewed research, blog posts, briefings, and websites. The search for grey literature consisted of two stages. First, a broad Google search was conducted using the same search terms and the first 10 pages of results were reviewed. Second, we hand‐searched the websites of major research‐active health and social care charities in the United Kingdom (e.g., Cancer Research UK and Alzheimer's Society), as well as UK Research Councils and funding bodies such as the National Institute of Health and Care Research (NIHR).

### 2.3 Study Selection

**Figure 2:**
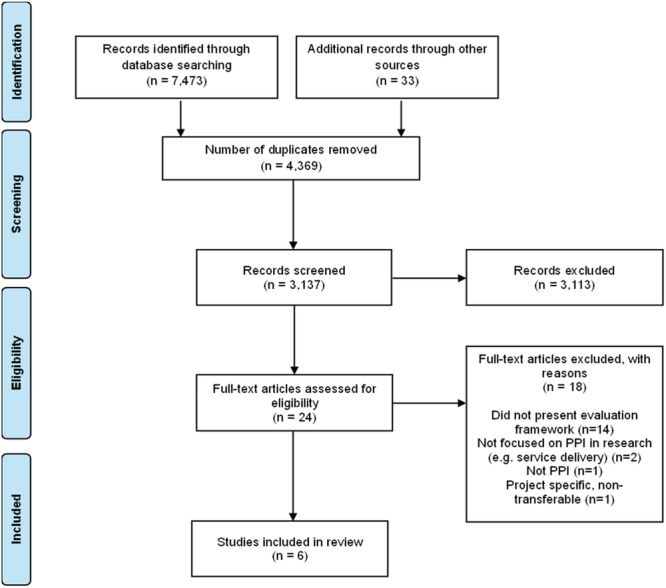
PRISMA flow diagram.

Once the search process was complete, duplicates were identified and removed using an online deduplication tool (https://sr-accelerator.com). The search of the electronic databases and grey literature generated 3137 records for screening after deduplication (Figure 2). These records were imported into screening software (https://new.rayyan.ai) for the first stage of screening. Two members of the team (RH and DB) conducted the initial title and abstract screening, and two other members of the team (AM and AAF) reviewed any disagreements, as well as all those included by RH and DB.

The inclusion and exclusion criteria for the review were pre‐established and modified through an iterative process as the searches progressed. A summary of these criteria is provided in Table 1. We included empirical studies (qualitative, quantitative, and mixed‐methods research), as well as review papers. Articles not focused on PI in health and social care research were excluded. As outlined earlier, we defined PI as a partnership where research is conducted ‘with’ or ‘by’ the public, rather than ‘to’, ‘about’, or ‘for’ them. Approaches such as Participatory Action Research were excluded because they can be considered research methodologies rather than PI. Articles from any country were eligible if the full text was written and available in English.

**Table 1:** Inclusion and exclusion criteria.

Inclusion	Exclusion
Peer‐reviewed empirical studies & review articles Non‐peer reviewed reports, blog posts, newsletters	Conference abstracts, dissertations, books, essays, audio accounts
Published between 2000 and 2024	Published before 2000
Any country of origin but full‐text available in English	Language other than English
Health and social care research	Other disciplines
Frameworks evaluating PI in research	Frameworks evaluating public engagement, public involvement in non‐research contexts, or participatory methodologies
An evaluation framework as defined by Nilsen [10]	Evaluation tools not meeting the definition of a framework
Frameworks focused on evaluating the quality of PI	Broader frameworks e.g. those evaluating the outcomes or impacts of PI
Frameworks that are transferable and can be used by other research teams	Frameworks not transferable to other research projects/activities
Sources focused on the development of a PI evaluation framework	Sources that do not present the development of the framework

Articles that did not use an evaluation framework were also excluded. To determine what counted as a framework, we drew on Nilsen's [10] definition, where a framework is considered “a structure, overview, outline, system or plan consisting of various descriptive categories, e.g., concepts, constructs or variables, and the relations between them that are presumed to account for a phenomenon”. Building from this, Nilsen [10] describes an evaluation framework as one that presents a structure for evaluating specific phenomena, in this case, PI quality.

Included frameworks also had to be transferable to other research projects or activities in order to have utility for other research teams, so frameworks that were project‐specific were excluded. For example, one study we identified had developed a questionnaire‐based tool to measure PI quality in their study. As this tool was based on their study, the questionnaire items were related to project specific activities and involvement. As a result, the tool in its current form would not have utility beyond the context in which it was developed.

During screening, we distinguished quality‐focused frameworks from broader PI evaluation tools by applying the following criteria. Included frameworks had to: (i) explicitly conceptualise or define quality (or related constructs such as “good,” “meaningful,” or “effective” involvement) and (ii) provide criteria, domains, or reflective questions that enable appraisal of the process of involvement itself. Frameworks and tools that primarily assessed the impact, outcomes, or value of PI (e.g., effects on research relevance, recruitment, dissemination, or participant experience), or that focused on documenting PI activities without explicit quality criteria, were excluded.

As the focus of the review was to identify and compare evaluation frameworks, we decided to focus on articles that presented the development of a framework. This strategy ensured that there was sufficient information provided regarding the framework's development and its theoretical underpinnings. Where an article used an existing framework but did not describe its development, the original article was sought out from the reference list for inclusion.

### 2.4 Charting, Collating and Summarising the Data

To select and record relevant information from the included articles, a data extraction sheet was developed. The purpose and aims of the scoping review were used to guide decisions about which data should be extracted. Study related information was extracted, including country of origin, year of publication, etc. Following pilot testing and discussions as a team, it was agreed that we wanted to capture information on both the development and application of the frameworks, as well as their broader relevance and utility.

The exact data items that were extracted evolved iteratively as extraction progressed. Information related to the following categories was collated:

- The aim of the framework.
- How it was developed.
- How it is delivered.
- Its reach (beyond the setting or scope in which it was developed).
- The involvement of public contributors in development and/or delivery of the framework.
- The approach to evaluation.
- The evaluation criteria used and any theoretical underpinnings.

Articles that were included following title and abstract screening were then subjected to full‐text screening, and relevant information was charted using the data extraction sheet. This process was completed by RH and DB, and extraction outcomes were then discussed with AM and AAF. Twenty‐four articles underwent full‐text screening, and six were included in final review (Figure 2).

The information collated using the data extraction sheet was summarised in line with the aims and objectives of the scoping review. To present the results, the domains within the data extraction sheet were developed to best present the key features of the frameworks.

## 3 Results

### 3.1 Characteristics of the Included Papers

Included articles were published between 2010 and 2023. All articles were based in the United Kingdom, aside from one, which was the result of a collaboration between international authors. Five out of six articles were identified from academic databases and one from grey literature searching. Six evaluation frameworks were identified from the included sources:

- CUBE.
- INSIGHT.
- PEQG (Patient Engagement Quality Guidance tool).
- PiiAF (Public Involvement Impact Assessment Framework).
- PIRIT (Public Involvement in Research Impact Toolkit).
- QIF (Quality Involvement Framework).

All identified frameworks were developed within an academic (university) context. They were created across a range of contexts and disciplines, including broader fields such as health and care research and health services research, as well as more specific fields such as cancer research and medicine development. Further detail on each framework is provided in Tables 2 and 3.

### 3.2 Aim of Frameworks

All of the frameworks identified shared a common same aim and purpose, to advance quality and champion best practice in PI. The exact focus of how that should be done, however, varied across the frameworks. The INSIGHT framework focuses on recognising and incentivising quality improvement at an organisational or departmental level. In contrast, frameworks such as PiiAF and PIRIT focus on supporting researchers to assess the impact of PI at a project level. PEQG combines these objectives, aiming to improve and assess quality and impact in ongoing or completed projects. The CUBE framework, on the other hand, is more theoretical and aims to describe the fundamental elements of successful PI, which can then be used to map research activities against it. QIF adopts a more general focus, supporting research teams by providing a structured approach to reflecting upon and reporting on quality in PI.

### 3.3 Method of Framework Delivery

The approach to evaluation methods also varied across the frameworks. QIF uses a questionnaire with a 5‐point Likert scale, derived from the theoretical model developed by the authors, with items divided across domains. The questionnaire is then completed by researchers and public contributors who report their perspectives on the perceived quality of PI activities. PiiAF and PIRIT take a different approach, encouraging research teams to develop their own plans and metrics of quality based on the nature of the project and the PI activities being undertaken. PIRIT consists of two elements: an impact Planning Tool and a Tracking Tool. These can be used separately or together, and alongside other frameworks. PiiAF involves an exercise to identify PI impacts specific to the research at hand, followed by the development of an impact assessment plan.

Both CUBE and PEQG draw on models encompassing multiple criteria that underpin quality. These models can then be used by researchers and public contributors to map their PI activities against the criteria. CUBE is often applied through a workshop, where public contributors are asked to position where they think their involvement lies across four evaluation dimensions. The result is a visual representation of PI that highlights where things went well, and where improvements are needed. The PEQG, on the other hand, includes two resources: one for planning stages, and one for ongoing or completed projects. The resources are based around the framework's seven quality criteria and encourage researchers to articulate how their PI activities meet each criterion, in what way, and within what timeframe.

The stage at which evaluation takes place also differed across frameworks. While some were designed for use during research planning, others could be applied regardless of research stage (Table 2).

**Table 2:** Summary of frameworks.

Source reference	Framework	Aim	Framework delivery	Evaluation constructs	Public involvement	Research stage
[11]	CUBE	To describe the fundamental elements for successful knowledge exchange, against which PI activities can be mapped and analysed.	Workshops with public contributors to map their experiences of involvement across different evaluation dimensions.	Weak voice/strong voice, one way to be involved/many ways to be involved, organisation's concerns/public concerns, and organisation changes/organisation resists change.	The framework was workshopped with three separate PI advisory groups.	Used in both the planning stages and across process of research.
[12]	INSIGHT	To recognise and incentivise quality improvement in PI on an organisational or departmental level.	Quality rating framework based on UKSPI‐derived quality indicators. Assessment uses appreciative inquiry approach.	Quality matrix based on four levels of involvement (Welcoming, Listening, Learning, Leading) for each of the six UKSPI.	Co‐produced using Task and Finish groups with public contributors involved across all stages and as co‐investigators. Public members involved in assessment panels.	Evaluates PI activities on an organisational level, so likely to cover various projects across stages.
[13]	PEQG	To improve and assess PI quality and impact in medicine development projects.	Research projects/activities are mapped against seven quality criteria.	Shared purpose, respect and accessibility, representativeness, roles and responsibilities, capacity and capability, transparency and communication, and continuity and sustainability.	Public contributors inputted through multi‐stakeholder working groups and a public consultation.	Can be used prospectively or retrospectively assess quality at planning, monitoring, and for completed projects.
[14]	PiiAF	To enable researchers to identify and assess the ways in which PI has impacted on their research.	Consists of two parts. 1) Identifying PI issues pertinent to research project, 2) development of impact assessment plan.	Encourages reflection in relation to four elements: values associated with public involvement, approaches to public involvement in research, research focus and study design, and practical issues shaping public involvement in research.	Public contributors were involved via a Public Advisory Group, an Advisory Network, and as co‐investigators.	Intended to be used in the research development process but can be used while research is ongoing.
[15]	PIRIT	To support researchers in tracking public contributions and the difference they make to the research.	Consists of two elements: the PIRIT Planning Tool and the PIRIT Tracking Tool. These can be used separately or together, and alongside other assessment frameworks.	Draws on UKSPI: Communications, Working Together, Inclusive Opportunities, Impact, Governance, Support and Learning.	Co‐developed by public contributors and researchers through meetings and working groups.	Consists of planning tool and tracking tool that can be used at the beginning and during research projects.
[16]	QIF	To support researchers by providing topics for reflection and a structure by which to report on their experiences of working together.	Questionnaire consisting of 31 items measured on a 5‐point Likert scale. Completed by public contributors and researchers.	Personal factors, e.g., feeling valued, and research factors, e.g., organisational support.	No PI activities reported.	Not specified but some questionnaire items featured in the article require retrospective knowledge of the research activities which limit use in development stages.

INSIGHT also draws on a model of quality featuring several criteria like CUBE and PEQG. Where INSIGHT differs is in its method of delivery, operating as a Quality Recognition Scheme and National Quality Awards Programme. Organisations are assessed by trained assessors (including public contributors) against the INSIGHT quality indicators (based on the UKSPI) and are given a rating (quality mark) for their PI activities. The assessment takes a strengths‐based appreciative inquiry approach recognising organisations' good practice, and incentivising quality improvement through a report that provides recommendations.

### 3.4 Evaluation Constructs

Many of the articles developed unique evaluation criteria during the creation of the framework. Conceptualisations of quality and best practice criteria varied across framework evaluation constructs, and these are summarised in Table 3.

**Table 3:** Conceptualisation of quality and definition of ‘best practice’ for each framework.

Tool	Conceptualisation of quality	Best practice criteria
CUBE	Hierarchical: categorisation of quality level (binary) for each of four quality parameters	Higher of the two levels for each parameter
INSIGHT	Hierarchical: categorisation of quality level (four levels) for each of six quality parameters	Higher of/improvement in the four levels for each parameter
PEQG	Value‐based indicators: reflections on seven quality criteria	How well the research is perceived to reflect the quality criteria
PiiAF	Value‐based indicators: reflections on four elements of quality	How well the research is perceived to reflect the quality criteria
PIRIT	Value‐based indicators: reflections based on the six UKSPI	How well the research is perceived to reflect the quality criteria
QIF	Hierarchical: on categorisation of quality level (5‐point Likert scale) for each of 31 questions	Higher Likert score

In creating their evaluation criteria, CUBE built upon the existing theories of knowledge exchange to develop four dimensions measured on a rising scale. One example is the strength of the public voice during their involvement, ranging from ‘weak’ to ‘strong’ (Table 2). This shares similarities with the criteria used in the INSIGHT framework, referred to as ‘quality indicators’. The indicators are hierarchical in nature, measuring the extent of the public's involvement within research activities. The INSIGHT framework consists of four ‘levels’ of involvement, against which organisations can be rated in relation to each of the six domains of UKSPI. These levels range from ‘Welcoming’, which relates to organisations welcoming members of the public to contribute to research, to ‘Leading’, where members of the public have a leadership role within research, or the team shows leadership in promoting PI quality outside their organisation. These criteria were co‐produced and derived from an integration of a framework developed by Expert Citizens, a Community Interest Company in Stoke‐on‐Trent, and the UKSPI [5].

PEQG takes a similar approach to that of INSIGHT, combining existing principles from NIHR INVOLVE [17] with the views of public contributors and insight generated from working groups recommendations. From this process, seven core principles were developed to help standardise PI, evaluate the quality of PI in existing projects, and document and share outcomes more consistently. The criteria are not hierarchical or dimensional and instead aim to delineate what they believe to be the core components of successful PI. Criteria include respect and accessibility, transparency and communication, and shared purpose (Table 2).

QIF draws on ideas around power and empowerment to construct a model of quality involvement. The model is divided into two domains: Personal Factors (e.g., a sense of being empowered) and Research Factors (e.g., the presence of organisational structures to support PI). This approach aims to give importance to both the perspective of the individual and the conditions under which public contributors are involved with research.

The frameworks focused on evaluating quality in terms of impact take a different approach. Unlike the other frameworks, where the evaluation criteria are either fully or partially developed for the purpose of creating the framework, PIRIT draws entirely on existing guidelines without modification. The PIRIT tools are linked to the UKSPI, and researchers are encouraged to report their PI impact against it. At the other end of the scale, PiiAF does not prescribe specific quality indicators. Instead, it encourages research teams to reflect on evaluating impact in ways that are relevant and appropriate to individual projects. It does, however, provide four constructs pertinent to PI impact as starting points for reflection, such as values associated with PI (Table 2).

### 3.5 Public Involvement in Framework Development and Delivery

There were varying levels of PI reported across the articles. Members of the public contributed to the development of the frameworks in a variety of ways (Table 2). PI activities included multi‐stakeholder working groups, task‐and‐finish groups, advisory groups, and workshops. Some articles explicitly mentioned public contributors being involved in a leadership capacity. The PIRIT research team, for example, included three co‐investigators who were public contributors. In contrast, QIF made no reference to PI in relation to the development of their framework. In terms of the diversity of public contributors involved in developing the frameworks, little information was provided within the articles. However, INSIGHT describes a partnership with Expert Citizens, a lived‐experience‐led community group focused on social disadvantage.

The extent to which public contributors were proposed to be involved in the delivery of the frameworks also varied. For the INSIGHT framework, public contributors have a key role as assessors and therefore are a central part of how the framework is delivered. While PIRIT and PiiAF encourage public contributors to be involved in the development and execution of impact plans and/or assessments, it is not a core component as such. While there seems to be no reason why public contributors could not be involved in the delivery of the PEQG, it is not reported within the article. Furthermore, the other frameworks arguably lean more towards public contributors as participants in the evaluation process rather than as partners; for example, through participation in CUBE workshops, or completion of a QIF questionnaire.

## 4 Discussion

This scoping review identified six frameworks developed to assess the quality of PI activities: INSIGHT, CUBE, PiiAF, PIRIT, QIF, and PEQG. To our knowledge, this is the first review focused specifically on identifying and comparing frameworks that evaluate the quality of PI in health and social care research. Analysis of the frameworks demonstrated a common goal to promote PI excellence, but with varied approaches in their development and delivery, conceptualisations of quality, and role in the research process.

### 4.1 Quality in PI

The way PI quality is conceptualised within the identified frameworks varies significantly. Several frameworks, such as INSIGHT, CUBE, and QIF, involve a hierarchical understanding of quality, often aligning with levels of involvement or empowerment (e.g., “Welcoming” to “Leading” in INSIGHT). Others, however, define quality more broadly through values‐based indicators such as respect, transparency, and shared purpose (e.g., PEQG, PIRIT).

This distinction raises questions around how the quality of PI activities should be assessed. For example, whether quality should be determined by the extent to which public contributors are involved (i.e., the depth of their involvement) or by the quality of the involvement process, regardless of how much decision‐making power is shared. Frameworks that equate deeper involvement with higher quality may help to reduce tokenism in PI by encouraging research teams to work with public contributors more fully. Similarly, an evaluation matrix with incremental levels may also incentivise quality improvement, as research teams and organisations may be encouraged conduct PI at a ‘higher’ level. However, such approaches risk overlooking the contextual needs of individual projects. For instance, some public contributors may not wish to have a leadership role.

It is important to consider whether the impact of PI on research outcomes should be equated with quality. As this review illustrates, measurement of the quality of PI itself remains variable, and its linkage to research quality will therefore also vary. Defining “impact” is challenging, and different frameworks emphasise different purposes. PIRIT and PiiAF focus on tracking the difference that PI makes to research, whereas other frameworks prioritise subjective measures, such as the experiences of public contributors and the extent of their involvement.

Evaluations also differ in whose perspective is considered; some foreground researcher‐defined outcomes, while others emphasise the views of public contributors. PI in the evaluation itself can further shape how quality and impact are assessed, for example through defining quality criteria, collecting data, or interpreting findings.

While all frameworks aim to strengthen PI in research, objectives for defining research impact remain challenging. Short‐term measures, such as grant success, are limited and subject to confounding factors, whereas patient‐focused measures, such as recruitment rates, appear more promising. Longer‐term outcomes, including impacts on patient health, remain difficult to demonstrate. Nevertheless, despite these challenges, there is widespread acceptance of the benefits of high‐quality PI, and the use of these tools is likely to have a positive effect on both quality and impact.

### 4.2 Strengths and Limitations of Frameworks

Each of the frameworks identified has strengths and limitations depending on context and intended use. INSIGHT, for instance, is unique in its organisational application, offering quality ratings, independent review panels, and formal recognition through awards. Through its use of quality ratings and awards, INSIGHT may incentivise improvements in PI by encouraging institutions to enhance their quality rating. In addition, its use of independent review panels provides impartial evaluation, supporting greater standardisation and comparability in assessing PI quality. However, its focus on institutional‐level assessment may obscure insights from individual projects or public contributors' experiences.

In contrast to INSIGHT, both PIRIT and PiiAF offer greater flexibility and adaptability to specific research contexts, enabling researchers and public contributors to define their own metrics of quality and impact. These frameworks may be especially useful where more rigid assessment may not be suitable due to the context of the research or involvement activities. That said, it can be argued this flexibility can come at the expense of comparability across studies and may challenge efforts to standardise quality in PI. Similarly, both PIRIT and PiiAF are focused on quality as impact which, as discussed earlier, means there is a slightly narrower focus when compared to other frameworks.

CUBE, with its interactive visual mapping and experience‐focused design, is valuable for capturing qualitative and relational aspects of PI. The dialogical nature of CUBE encourages reflection and amplifies the voice of public contributors and can be used to gain feedback at any stage of research to make improvements. However, similarly to INSIGHT, the use of set domains reduces the ability to make context‐specific adaptations, although the use of consistent domains allows for cross‐study comparisons.

QIF's structured questionnaire format provides a practical tool to capture the perspectives of those involved in PI. The distinction between ‘personal’ and ‘research’ factors provides a useful way of considering both the experiences of individuals but also the wider structures in which PI takes place. One limitation of QIF is that there was no description of public contributors' involvement in the development of the framework which suggests there was none. Similarly, the use of structured questionnaires in the framework's delivery could make public contributors feel like participants within the evaluation rather than active collaborators.

Like PIRIT and PiiAF, a key strength of the PEQG framework is its utility for both prospective and retrospective evaluation, comprising a resource for planning as well as one for ongoing or completed projects. Drawing on seven values‐based indicators, PEQG provides research teams with guiding principles to consider when appraising the quality of their PI activities. As these principles are broad and non‐hierarchical, they also allow for consideration of contextual factors. This approach may be particularly beneficial for those looking for a less prescriptive approach but who have less experience of PI and so may not feel confident developing their own evaluation metrics.

It is acknowledged that most of these tools were developed in the United Kingdom. This may reflect the limitation in focusing on terms for PI most frequently used in the United Kingdom. However, we feel that the potential utility and adaptability each of these tools to other countries remains strong due to their broad‐based themes. INSIGHT, for example, was developed with different sectors (academic, healthcare, pharmaceutical sector, third sector) and potential global utility in mind.

### 4.3 Recommendations for Framework Selection

The decision as to which framework(s) to use in a given situation depends on a series of factors. Based on our findings, we have provided researchers with suggestions on which framework(s) they might consider based on the strengths and limitations of each framework (Table 4). We advise researchers to consider the following.

**Table 4:** Strengths and limitations of PI quality frameworks.

	Level		Stage			Prescriptiveness		Incentivisation		Validation	
Framework	Project	Organisation	Planning	Monitoring	Retrospective review	Framework quality matrix	Project‐derived metrics	Incremental levels	PI awards	Independence	Acceptability
INSIGHT	±	Y	±	±	Y	Y	N	Y	Y	Y	N
PiiAF	Y	N	Y	Y	Y	N^a^	Y	N	N	N	Y
PEQG	Y	±	Y	Y	Y	Y	N	N	N	N	N
CUBE	Y	±	Y	Y	Y	Y	N	Y	N	N	Y
QIF	Y	±	N	N	Y	Y	N	Y	N	N	N
PIRIT	Y	N	Y	Y	Y	N^a^	Y	N	N	N	Y

*Note:* “±” could be adapted for this purpose.

^a^ While not prescriptive, these frameworks provide domain guidelines to guide development of project‐specific metrics.

#### 4.3.1 Level

If the goal is to assess PI at the project level, most frameworks (e.g., PIRIT, PiiAF, CUBE, QIF, PEQG) will be suitable. For evaluating PI across an entire organisation or department, INSIGHT is the most appropriate, though its quality matrix could be adapted for use at the project level.

#### 4.3.2 Research Stage

When planning PI, QIF may be less useful as some questions require retrospective data. For monitoring during a project, PIRIT's tracking tool, along with PiiAF, CUBE, and PEQG, offer suitable options. For retrospective reviews, any of the frameworks can be used effectively. Selection should be based on whether the focus is forward‐looking planning, real‐time monitoring, or reflective assessment.

#### 4.3.3 Prescriptiveness

For projects seeking predefined quality descriptors, frameworks such as INSIGHT, PEQG, CUBE, and QIF provide structured matrices. For projects requiring tailored metrics to fit specific contexts, PiiAF is more flexible, offering domains as a starting point for developing project‐derived measures. PIRIT sits in between, drawing on UKSPI as guidance while focusing on impact.

#### 4.3.4 Incentivisation

Where the aim is to encourage improvement in PI quality, frameworks with incremental quality levels such as INSIGHT, CUBE, or QIF may be advantageous. INSIGHT goes further by using appreciative inquiry, providing recommendations for improvement, and recognising excellence through awards, supporting institutional change as well as project‐level growth.

#### 4.3.5 Validation

For contexts where independent or external validation of PI quality is important, INSIGHT is the strongest option as it involves review panels with both PI professionals and public contributors. Where reflective practice and self‐assessment are sufficient, the other frameworks may be more appropriate. The choice depends on how much weight external verification carries within the discipline.

Researchers are advised to select evaluation frameworks that best align with the aims and context of their project, considering factors such as level, stage, prescriptiveness, incentivisation, and validation. Researchers may find it useful to begin by deciding whether to adopt a hierarchical conceptual framework or a more reflective, values‐based approach (Table 2). This decision can be guided by the type of project, the intended outcomes, and the needs and preferences of public contributors. Careful alignment will help ensure that PI evaluation is both meaningful and fit for purpose, rather than applying a one‐size‐fits‐all approach.

### 4.4 Strengths and Limitations of the Review

We recognise that the varying definitions and terminology around PI presents significant challenges. Boivin et al. [3, p. 1] state that “the language, definitions, and goals of PI vary between stakeholders, cultures, and countries”. A key limitation of this scoping review is that it focused specifically on the concept of PI and did not include public engagement as a search term. PI is a well‐established, policy‐relevant term in the UK health and social care research context [18], whereas public engagement is used more widely internationally and across sectors, including higher education and the VCSE sector, to describe active, two‐way collaboration with members of the public and people with lived experience [19]. Other related terms, such as consumer involvement, consumer engagement, and community engagement, were also not captured. Many of these terms represent overlapping but conceptually distinct approaches that vary in meaning, application, and underlying principles across disciplines and countries. Expanding the search to include these terms would have substantially increased the scope and heterogeneity of the literature and was beyond the remit of this review.

Similarly, our decision to exclude articles that did not describe an evaluation framework meant that we did not include papers describing the UKSPI itself or reporting tools such as GRIPP2 [20], which may also support high‐quality PI. However, the UKSPI were not intended to be used as a measure of PI quality and the GRIPP2 tool focuses solely on describing PI in publications and reports.

As the aim of the review was to map and explore frameworks within the existing literature, no formal critical appraisal of included studies was conducted. As a result, it is not possible to comment on the methodological robustness of research underpinning the frameworks identified.

We acknowledge that there was no PI in the early stages of the scoping review process, such as in the development of the research questions. This was restricted by lack of funding for the project, and we have attempted to address this, by strengthening public contribution during the later stages of the work. A public contributor (AMe) was invited to join the team as a co‐author during manuscript development and revision. In this role, (AMe) undertook a retrospective critical review of the review design, inclusion and exclusion criteria, interpretation of findings, and the conceptualisation of “quality” in PI. He provided detailed written and verbal feedback on the manuscript and also contributed to the revisions made in response to peer review.

While this retrospective involvement does not fully substitute for PI in the initial design of the scoping review, it has strengthened the reflexivity, clarity, and practical relevance of the revised manuscript. In future studies, efforts should be made to involve public contributors from the outset, including in shaping research questions and the review design. In the absence of research grant support, targeted funding should be sought to enable early involvement of public contributors.

The review identified a number of expected frameworks (which acted as a sense check of the search) as well as some additional ones. Our analysis of the six frameworks provides guidance for researchers and organisations in health and social care research on selecting the most appropriate framework for different contexts. We hope this guidance will support research teams to undertake meaningful evaluation of PI that is consistent with the context in which they are working.

### 4.5 Future Research

Given this review's conceptual focus on PI frameworks, future research could explore the overlap and distinctions in approaches to evaluation between PI and related terms such as public engagement, co‐design, and co‐production. This would help identify transferable learning across sectors such as higher education and the voluntary and community sector, as well as across international contexts.

Further studies could also examine how PI has shaped the development of evaluation frameworks and assess the impact of this involvement on framework design, relevance, and usability. In addition, research is needed on the practical application of these frameworks, including the organisational settings in which they have been used and their impact across different research contexts. Addressing these areas would strengthen understanding of PI evaluation and inform the development, refinement, and selection of frameworks for use across diverse research contexts.

## 5 Conclusion

This review presents the first comparative analysis of frameworks designed to assess the quality of PI in health and social care research. By mapping their strengths, limitations, and contexts of use, we provide practical guidance for researchers, institutions, and public contributors seeking to enhance PI practice. Crucially, there is no one‐size‐fits‐all solution. The most suitable framework will vary depending on the goals of evaluation and the specific requirements of the project or organisation.

## Author Contributions

**Rose Hutton:** conceptualisation, writing – original draft, methodology, writing – review and editing. **Alice Moult:** conceptualisation, writing – original draft, methodology, writing – review and editing. **Dereth Baker:** conceptualisation, methodology. **Anthony A. Fryer:** conceptualisation, methodology, writing – review and editing, writing – original draft. **Andrew Meakin:** writing – review and editing, writing – original draft. **Opeyemi Babatunde:** writing – review and editing, writing – original draft.

## Conflicts of Interest

Three of the authors (A.A.F., A.Me. and O.B.) are, or have previously been, involved in the development and ongoing research of the INSIGHT evaluation framework discussed in this review.

## Supporting information

Supporting File

## Data Availability

Data sharing not applicable to this article as no datasets were generated or analysed during the current study. Additional materials are included as Supporting Information S1: documents.
