# Supplementary material for: Frameworks to Evaluate the Quality of Public Involvement in Health and Social Care Research: A Scoping Review
Source: Health Expect. 2026 May 16;29(3):e70666. doi: 10.1111/hex.70666 (PMC13179752; doi:10.1111/hex.70666)
Supplement: Supplementary file 1 — Supporting File [file HEX-29-e70666-s001.docx]

**Evaluating quality in public involvement in health and social care research**

**Data extraction form**

| **Date extracted** |  |
| --- | --- |
| **Extracted by** |  |
| **Author(s)** |  |
| **Title** |  |
| **Publication year** |  |
| **Journal/source** |  |
| **Country** |  |
| **Peer reviewed?** |  |
| **Type of publication** |  |
| **Notes** |  |

| **Domain** |  | **Description** | **Page number** | **Notes** |
| --- | --- | --- | --- | --- |
|  | **Name of framework/tool** |  |  |  |
| **Purpose/Incentivisation** | **Aim/purpose of framework/tool** |  |  |  |
| **Reach/Flexibility** | **Who is it intended for? (e.g. what is the flexibility/reach)** |  |  |  |
| **Primary source/reach** | **Is the development of the framework/tool the focus of the article/source?** |  |  |  |
| **Co-production/drivers** | **How was framework/tool developed?** |  |  |  |
| **Incentivisation** | **Does framework/tool incentivise quality of PI? If so, how** |  |  |  |
| **Co-production**  **Diversity** | **Were patients/public involved in development of framework/tool? (If so, how)**  **Inc. diversity of involvement** |  |  |  |
| **Co-delivery**  **Diversity** | **Were patients/public involved in delivery of framework/tool? (If so, how)**  **Inc. diversity of involvement** |  |  |  |
| **Evaluation**  **Strength- or deficit-based** | **Have the authors evaluated the framework/tool? If yes, how has it been evaluated?** |  |  |  |
| **Evaluation** | **Has the tool/framework been piloted? If yes, with who?** |  |  |  |
| **Reach/Flexibility** | **Area(s) of health and social care** |  |  |  |
| **Purpose (might overlap with aims)** | **Stage of research cycle it applies to (e.g. reporting, grant development etc)** |  |  |  |
|  | **Definition of PI used** |  |  |  |
